# The Drugs of Sleeping Sickness: Their Mechanisms of Action and Resistance, and a Brief History

**DOI:** 10.3390/tropicalmed5010014

**Published:** 2020-01-19

**Authors:** Harry P. De Koning

**Affiliations:** Institute of Infection, Immunity and Inflammation, University of Glasgow, Glasgow G12 8TA, UK; Harry.de-Koning@glasgow.ac.uk; Tel.: +44-141-3303753

**Keywords:** sleeping sickness, human African trypanosomiasis, trypanosoma brucei, drugs, drug resistance, history

## Abstract

With the incidence of sleeping sickness in decline and genuine progress being made towards the WHO goal of eliminating sleeping sickness as a major public health concern, this is a good moment to evaluate the drugs that ‘got the job done’: their development, their limitations and the resistance that the parasites developed against them. This retrospective looks back on the remarkable story of chemotherapy against trypanosomiasis, a story that goes back to the very origins and conception of chemotherapy in the first years of the 20 century and is still not finished today.

## 1. Introduction

The first clue towards understanding drug sensitivity and, conversely, resistance, in human African trypanosomiasis (HAT) is that most drugs are very old and quite simply toxic to any cell—if they can enter it. That places the mechanisms of uptake at the centre of selectivity, toxicity and resistance issues for all the older trypanocides such as diamidines (e.g., pentamidine, pafuramidine, diminazene), suramin and the melaminophenyl arsenicals. Significantly, none of these drug classes, dating from the 1910s to the 1940s, were designed for a specific intracellular target and even today identification of their targets has defied all attempts with advanced postgenomic, proteomic and metabolomic techniques—in short, they are examples of polypharmacology, where the active agent acts on multiple cellular targets. One might say they are non-specifically toxic. As such, resistance is unlikely to occur from mutations that change the binding site of a particular intracellular protein. Rather, the resistance mechanisms have been associated with mechanisms of cellular uptake and/or distribution. Some of the newer drugs have a more defined mode of action, and are selective at target level, but resistance to eflornithine, at least, is still associated with the loss of the *T. brucei* transporter that internalises it, rather than with the target enzyme. In the sections below I will examine these issues for each drug separately and show how resistance and treatment failure have changed clinical treatment of sleeping sickness and stimulated the development of the newer generations of drugs, culminating in the latest additions to the arsenal (fexinidazole, acoziborole) [1,2,3,4].

## 2. Diamidines

**Figure 1:**
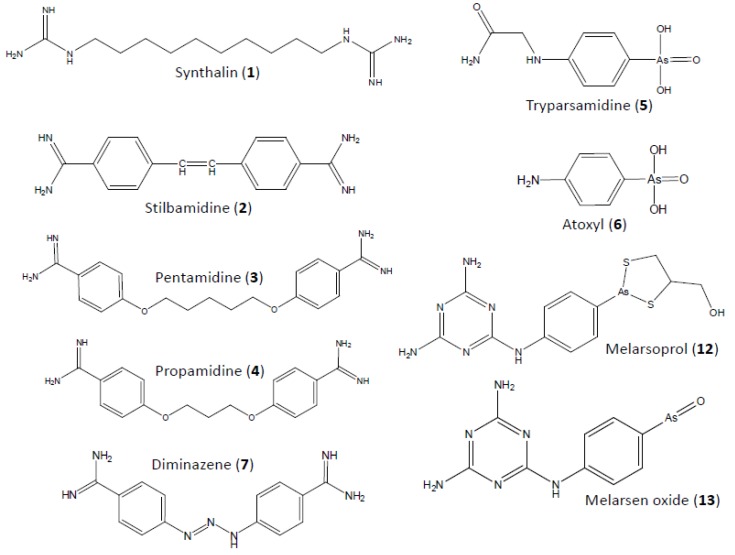

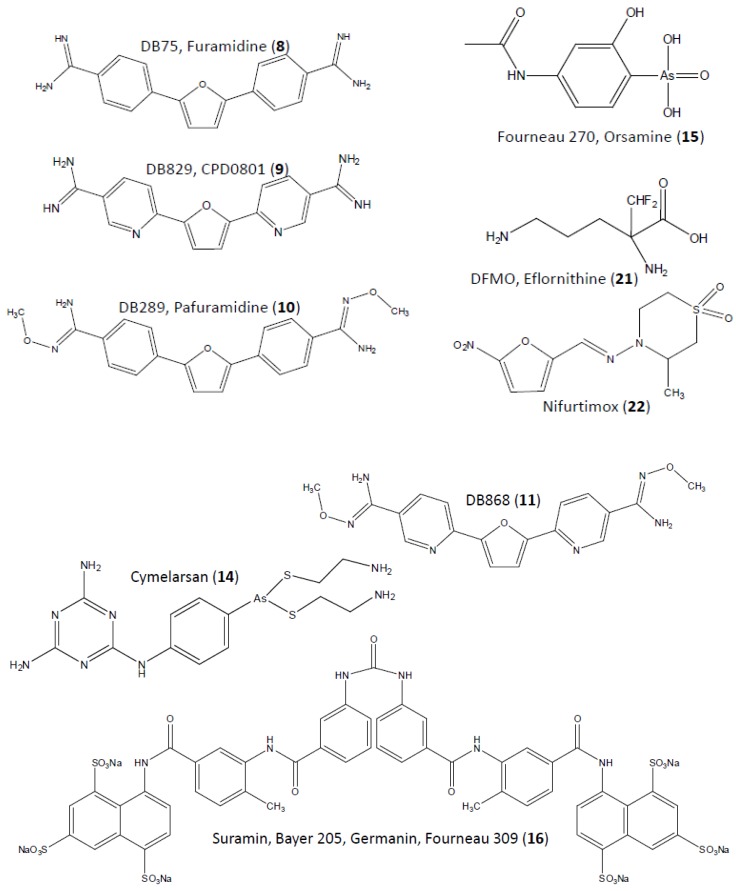

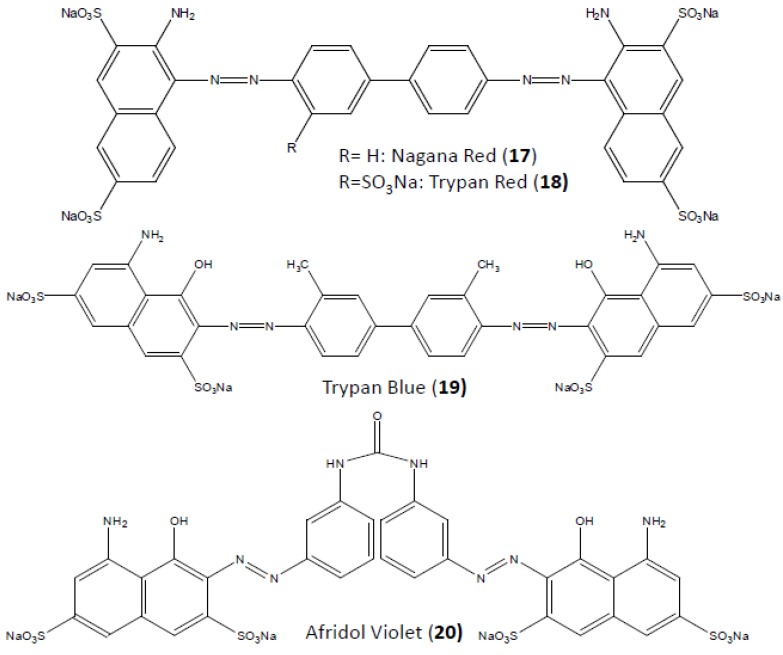
Structural formulas of trypanocides.

The development of the diamidines arose from the observations that advanced (animal) trypanosomiasis is often associated with hypoglycaemia [5,6] and trypanosomes metabolise glucose at a phenomenal rate. This suggested that the chemical induction of hypoglycaemia might be deleterious to trypanosomes in the bloodstream. Several groups tested insulin and other hypoglycaemia-inducing therapies against trypanosomiasis but with at best mild and variable success [7,8]. However, the synthetic insulin substitute synthalin (**1**; for structures see Figure 1) did have remarkable, curative trypanocidal activity [8,9] and, importantly, was not cross-resistant with the aromatic arsenicals used at the time, nor with suramin (“Bayer 205”) [10]. Although it was not immediately clear to what extent this could be attributed to effects on blood sugar levels, that question was rapidly settled by the trypanocidal effects of synthalin on ex vivo trypanosomes [11]. By 1939, Lourie and Yorke, in collaboration with A. J. Ewins of May & Baker Ltd, reported on a large series of new diamidine compounds, among them 4,4’-diamidinostilbene (stilbamidine, **2**) and 4,4’-diamidino,1,5-diphenoxy pentane (pentamidine, **3**) [12]. Stilbamidine was the most active compound—curative with 25–50 µg per 20 g mouse (1.25–2.5 mg/kg b.w.) and a therapeutic index of 30—closely followed by propamidine (**4**) and pentamidine, which displayed a slightly lower therapeutic index of 15. To appreciate the enormous advance this signified, these numbers need to be compared to the dramatically higher minimum curative doses for the aromatic arsenicals then in use: 1000 mg/kg for tryparsamidine (**5**) or 250 mg/kg for atoxyl (**6**), each with a therapeutic index of just two [12]! As stilbamidine appeared to induce adverse neurological sequelae in early clinical trials [13], it was abandoned and pentamidine became the drug of choice for early stage HAT, especially of the *gambiense* variety. The now exclusively veterinary analogue diminazene aceturate (“Berenil”, **7**) has also been used initially (and later sporadically) against HAT [14,15], but this practice has long been discontinued.

**Figure 2:**
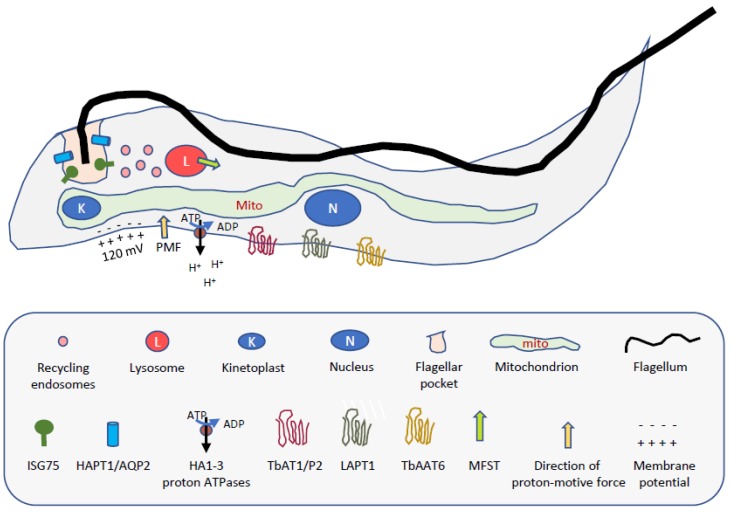
Schematic representation of a *T. brucei* trypomastigote, indicating some of the structures and proteins involved in the uptake or mechanism of action of trypanocides.

Diamidines are believed to be minor groove binders and as such bind to the DNA double helix, particularly targeting AT-rich sequences [16,17,18,19], impeding replication and transcription processes in the kinetoplast and/or nucleus. Usually, they accumulate strongly in the trypanosome’s single mitochondrion (and mitochondria of cancer cells [20]), the compartmentalisation of these dications being driven by the mitochondrial membrane potential and binding to the kinetoplast DNA (kDNA) (for a schematic of the trypanosome structure, see Figure 2). Indeed, fluorescent diamidines light up the kinetoplast within 1 minute of administration, a process that is much delayed in resistant parasites [21]. Thus, pentamidine is known to accumulate up to mM levels inside trypanosomes [22] and does not exit the cell when the extracellular drug is removed [23]. Furamidine (**8**) and its analogues reportedly accumulate to > 10 mM, associating strongly with kinetoplast and nuclear DNA [17,24]. Similar processes drive mitochondrial accumulation of other cationic trypanocidal agents including isometamidium [25], symmetrical compounds with choline-like head groups [26], furamidines [21,27,28], shielded bis-phosphonium compounds [29] and inhibitors of Trypanosome Alternative Oxidase (TAO) linked to a lipophilic cation [30,31]. Resistance to minor groove binders cannot occur via mutations in the target and the binding affinity does not need to be very high if the accumulation of the drug is to the high local concentrations reported. Thus, resistance is associated with the inability of the diamidine to reach its target, either by preventing its uptake into the cell altogether, or at least preventing its accumulation in the mitochondrion. The latter mechanism was demonstrated in pentamidine-resistant *Leishmania mexicana* parasites [32].

While pentamidine is used exclusively for the treatment of stage I (haemolymphatic) HAT, there have been reports of successes with ‘early late stage’ infections, where the parasite has crossed the blood-brain barrier (BBB) but not yet fully penetrated the brain parenchyma [33]. In particular, a study from 1996 reported a cure rate of 94% of early-late stage HAT patients with pentamidine [34]. Thus, pentamidine must be aided across the BBB by a transporter, and Sekhar et al. identified the Organic Cation Transporter 1 (OCT1) as responsible for the process [35], as previously reported in experiments with cell lines expressing all three human OCT isoforms [36]. The reason that pentamidine is not active against cerebral trypanosomiasis, then, is because it is actively extruded again from the CNS to the blood, by P-glycoproteins (P-gp), Multidrug Resistance-Associated Proteins (MRPs) or other extrusion transporters [37]; Yang et al (2014) later reported that pentamidine and the furamidines are not substrates for P-gps [38]. Other diamidines such as diminazene and furamidine are equally ineffective against cerebral trypanosomiasis [39,40,41], and the distribution of DB75, although readily detectable in-whole brain extracts, was limited to the cells lining the BBB and blood–cerebrospinal fluid (CSF) barriers as it did not penetrate the brain parenchyma [36]. 

However, DB829 (2,5-bis(5-amidino-2-pyridyl)furan; (**9**)), a close analogue of furamidine, did display remarkable efficacy against cerebral trypanosomiasis in mice and in vervet monkeys [42,43]. This was taken as evidence that DB829 is either recognized more efficiently than furamidine by a BBB transporter importing it into the CSF, or less efficiently extruded from the CSF by a P-gp-type transporter; both compounds have a similar pK_a_ and are dications at physiological pH, precluding any notion that they could simply diffuse across the barrier. However, the late failure of the clinical trials with pafuramidine (**10**), as a result of delayed nephrotoxicity in a small number of patients [40], also impeded clinical development of the all too similar DB829 and its prodrug DB868 (**11**).

The first resistance mechanism identified for the diamidines pentamidine and diminazene (the most-used drugs for early-stage HAT and for AAT, respectively) was loss of the P2 aminopurine transporter [44,45,46], which is encoded by the gene *TbAT1* [47]. Deletion of this gene did result in modest loss of in vitro pentamidine sensitivity in *T. b. brucei* bloodstream forms (and high-level resistance to diminazene [48]) but not to the extent that it would lead to clinical treatment failure. Indeed, Graf et al. [49] established that field isolates of *T. b. gambiense* from HAT patients that carry the *TbAT1* resistance allele were highly resistant to diminazene but only marginally less sensitive to pentamidine, compared to strains carrying the reference *TbAT1* allele. The *TbAT1^R^* allele contained several amino acid changes and a 1-codon deletion, compared to the reference. These mutations were systematically evaluated, separately and in groups, by expressing the various mutant forms in a *tbat1*-null strain of *T. b. brucei* [50]. Surprisingly, none of the single amino acid mutations changed pentamidine transport or sensitivity much, but the introduction of two and particularly three such changes at the same time all but disabled the transporter’s capacity for pentamidine uptake. The mutational studies were combined with homology modelling of the TbAT1 protein and produced a strong model of substrate binding, in which both aminopurines and pentamidine are bound via dual H-bonds with Asp140 and aromatic interactions with phenylalanines 19 and 316 [50]. This binding mode confirmed earlier binding models based on substrate binding energies with a large set of adenosine and diamidine analogues [51,52]. 

However, the transport of [3H]-pentamidine by *T. b. brucei* is inhibited only partially by P2/TbAT1 substrates adenine and adenosine, leading to the unambiguous conclusion that (an)other transporter(s) must also be involved [45,53]. Two additional transporters were identified, with K_m_ values of 36 ± 6 nM and 56 ± 8 µM (compare 0.43 ± 0.02 µM for TbAT1) and were accordingly designated the High Affinity Pentamidine Transporter (HAPT1) and the Low Affinity Pentamidine Transporter (LAPT1) [53]. Adaptation of the *tbat1*-null cell line to higher levels of pentamidine in vitro produced a strain, B48, that had lost HAPT1 activity and was 130-fold resistant to pentamidine in vitro (and was also highly resistant to melaminophenyl arsenicals, vide infra) [54]. This clonal strain had lost the HAPT1 transporter but retained normal LAPT function [54]; efforts to induce even higher levels of pentamidine and induce mutations in LAPT1 were unsuccessful [55]. The conclusion from this work was that the HAPT1 transporter was the most important contributor to pentamidine sensitivity in *T. brucei*, with LAPT playing only a minor role at therapeutically relevant drug concentrations.

A genome-wide screen for genes conferring pentamidine sensitivity with an RNAi library identified two loci, encoding for the plasma membrane proton ATPases HA1, HA2 and HA3, and for the aquaglyceroporins TbAQP2 and TbAQP3, respectively [56]. The involvement of the proton pumps indicates that pentamidine uptake is dependent on the proton-motive force or plasma membrane potential (as previously reported for procyclic *T. b.* brucei [53]), which the ATPases help maintain, but the latter RNAi ‘hit’ was harder to explain, as aquaporins are not known to engage in the uptake of relatively large molecules such as pentamidine, and certainly no cations. However, deletion of TbAQP2, but not of TbAQP3, resulted in a high-level of pentamidine resistance [57], and expression of TbAQP2 in *Leishmania mexicana* promastigotes, which are normally not very sensitive to pentamidine, increased their sensitivity ~40-fold and introduced a high affinity transport function that was indistinguishable from HAPT1 of *T. b. brucei* by K_m_ and inhibitor profile [55]. 

One possible explanation for the implausible uptake of dicationic pentamidine by an aquaporin is that pentamidine simply binds to the extracellular face of TbAQP2 and is internalised through endocytosis when the protein is internalised for turnover [58]. However, the rate of high affinity pentamidine transport in *T. brucei* procyclic cells is > 10-fold higher than in bloodstream forms [53,59], despite a much lower rate of endocytosis [60]. Moreover, although TbAQP2 is solely localised to the flagellar pocket in bloodstream forms [57], it is located all over the plasma membrane of procyclic forms, and the flagellar pocket is the sole site of endocytosis in trypanosomes [61]. For the same reasons, the high rate of pentamidine uptake in *L. mexicana* promastigotes expressing *TbAQP2* [55] seems incompatible with the endocytosis model. The alternative explanation is that pentamidine does in fact traverse the TbAQP2 channel, and this is explained by the unique changes to the selectivity filter motifs characteristic of aquaporins; these changes have the combined effect of making the pore much wider and removing the cation-excluding arginine residue [57,62]. This allowed docking of the stretched-out pentamidine in a low-energy conformation inside the pore of a model of TbAQP2 [62]. Much work has subsequently been done to supply definitive proof of either model, and this has definitively come down on the side of uptake through the TbAQP2 pore [63].

In summary, pentamidine resistance is principally associated with changes in *TbAQP2* through mutations, deletions and chimeric rearrangements with the adjacent *TbAQP3* gene. Interestingly, resistance can be bypassed with a nanotechnology formulation of pentamidine in chitosan nanoparticles coated with single domain nanobodies that specifically target trypanosomal surface proteins [64]. 

## 3. Melaminophenyl Arsenicals

Arsenic-based drugs were the very first treatments against sleeping sickness, starting with the use of inorganic sodium arsenite to treat animal trypanosomiasis or ‘nagana’ by David Livingstone in 1847 or 1848 [65] and David Bruce in 1895 [66]. Although neither of these pioneers achieved a full cure with this treatment (the animals eventually relapsed), it led to the development of the first organo-arsenic compound for sleeping sickness, atoxyl (**6**) as early as 1905, at the time of the major sleeping sickness epidemic in Central and East Africa [67]. However, atoxyl, meaning ‘non-toxic’ had severe side effects and was only active against early stage HAT, and was followed by tryparsamide (**5)** in 1919 [68]. Tryparsamidine was the first drug to be active against the late stage; however, it was only used against *gambiense* HAT and was considered of little use to either the early or late stage of the Rhodesian form [7,69,70]. A very similar compound, acetyl-*p*-amino-O-oxophenylarsenic acid, known as Fourneau 270 or Orsanine (**15**), was synthesised by Ernest Fourneau at the Institut Pasteur in Paris [71] and had very similar properties as tryparsamide [72,73]; indeed, it was claimed to have twice the selectivity index of tryparsamidine [74] and was in use for approximately 15 years [75].

The melaminophenyl arsenicals (MPA) replaced these earlier arsenicals, particularly tryparsamide, partly because melarsoprol (**12**) had better penetration into the central nervous system (‘rendered trypanocidal the cerebrospinal fluid of rabbits’ [76]) and tryparsamidine was poorly active against the early stage of the disease [14]. In fact, the MPAs were the first cure for late stage Rhodesian sleeping sickness, which had been considered incurable up to then [72]. More importantly yet, resistance to tryparsamide had developed and had become widespread throughout the early 1940s, to the point that the drug had become ineffective in most cases, particularly in French West Africa and Belgian Congo [77]; in 1947, 80% of new cases in Congo were reportedly resistant to what was then the only treatment for late stage HAT [7,14]. Moreover, resistance to both atoxyl and tryparsamide had been shown to be highly stable over prolonged periods, with Yorke and Murgatroyd concluding that it ‘persists indefinitely’, even when passed repeatedly through tsetse flies [78]. The MPAs were introduced in the late 1940s [14,69], despite reservations about their toxicity [79], largely driven by concerns about tryparsamide resistance. Extensive experience with MPAs in French West Africa was contained in a 1953 report by the Service Général d’Hygiène Mobile et de Prophylaxie de l’Afrique Occidentale Française [80] and concluded that the proposed “detoxified melarsen oxide”, Mel B (melarsoprol, “Arsobal”) was at least as toxic as melarsen oxide (**13)** itself but less active, and strongly recommended a return to melarsen oxide. However, the report also acknowledged that the MPAs as a class were a step forward and active against strains resistant to the older arsenicals. Ian Apted, in his comprehensive 1970 review of HAT treatments, states that melarsoprol, developed from melarsen oxide by reaction with Dimercaprol, also called British anti-Lewisite (BAL; antidote for arsenic poisoning) was less toxic than the original melarsen and melarsen oxide [72]. However, even after melarsoprol became the standard treatment for late stage sleeping sickness, the high level of severe adverse effects, with an estimated 10% of patients suffering from reactive encephalitis, half fatal [81,82] remained acceptable only for lack of alternatives. The introduction of an optimised 10-day administration schedule in 2005 [83] reduced the total amount administered and saved money but did not reduce the adverse effects significantly. A further and highly promising development was the proposal of melarsoprol cyclodextrin complexes as the first oral treatment of HAT [84]. In mouse models, this protocol appeared to be much safer, and it negated the need for intravenous administration of melarsoprol, as a caustic solution in propylene glycol. The melarsoprol cyclodextrin complex was awarded orphan drug status by the European Medicines Agency (EMA) in October 2012 [85] and by the U.S. Food and Drug Administration (FDA) in 2013. A protocol for phase 2 clinical trials of oral complexed melarsoprol in late stage *T. b. rhodesiense* HAT in Uganda was subsequently approved by the EMA (Peter Kennedy, personal communication). However, this has not yet been implemented for lack of funding, presumably because new human trials with arsenicals would be hard to get funding for, and NECT and fexinidazole came along as timely alternatives, at least for *gambiense* HAT (see below).

The discovery of the resistance mechanisms for the MPA melarsoprol (and its veterinary analogue cymelarsan (**14**)) is very similar, and parallel to that of the diamidines. Indeed, the phenomenon of pentamidine-melarsoprol cross-resistance (MPXR) was first reported almost 70 years ago [86,87], and at the time hypothesised to be due to the presence of similar motifs in the melamine-phenyl group and benzamidine moieties and thus ‘loss of permeability to, or adsorption affinity of, the melamine grouping in the melarsen-resistant strain may possibly prevent initial uptake of the amidine-type drugs’ as well [86]. This insight, based solely on cross-resistance patterns and a faint structural similarity, has now been well validated [88].

The association of MPAs with uptake via P2/TbAT1 was first discovered by Carter and Fairlamb in 1993, who found that out of a large number of biochemicals only adenine, adenosine and the transport inhibitor dipyridamole partly blocked the trypanocidal activity of melarsen oxide, and that an MPA-resistant strain had lost the function of one purine transporter, which they termed P2 [89]. This was later confirmed by other researchers [90], and the joint recognition motif for adenosine, diamidines and MPAs formally established [51,52,91]. As related in the previous section, the P2-encoding gene, *TbAT1*, was identified in 1999 and an allele bearing multiple polymorphisms was found to confer resistance [47]. A similar resistance allele was reported from Uganda in 2001 and was found more frequently in melarsoprol relapse patients than in those cured [92,93]. This was followed by experimental evidence that the deletion of *TbAT1* led to a loss in in vitro MPA sensitivity [48]. 

As reviewed elsewhere [94], concerns of melarsoprol treatment failure had been rising throughout the 1990s and early 2000s [95,96,97,98]. The confirmation of *TbAT1* resistance alleles, as well as the well-documented toxicity of melarsoprol [81] and the confirmation that the treatment failures could not be attributed to individual patients’ differences in melarsoprol pharmacokinetics and distribution [99], shifted first-line treatment in many centres, including Omugo in Uganda, to eflornithine monotherapy. Resampling of clinical isolates 4 years later no longer detected the *TbAT1^R^* allele in Omugo; in contrast, the mutant allele was readily amplified from patients, including five relapse cases, in the Moyo treatment centre, also in Northern Uganda, that had continued to use melarsoprol as the first-line drug [100]. Similarly, no *TbAT1^R^* alleles and no MPA-resistant isolates were found in a 2007 clinical study in South Sudan—an area that had also switched to eflornithine in 2001 after high melarsoprol relapse rates [101]. However, in the latter study it remained unproven whether the *TbAT1* mutations had disappeared after melarsoprol treatment had been largely discontinued or that, alternatively, the treatment failures had not been due to *TbAT1* mutations in the first place, as no sampling had been done before the switch to eflornithine. Conversely, the Ugandan study had not tested the *TbAT1^R^*-bearing isolates MPA for sensitivity in vitro or in a controlled animal model, and thus the link of clinical melarsoprol failure and *TbAT1* mutations has remained formally unproven, although highly plausible. Specifically, it could not be ruled out that additional factors (whether patient- or trypanosome-related) also played a role.

Meanwhile, Bridges et al. [54] showed that MPA resistance was much higher in laboratory strains that had functionally lost both the TbAT1/P2 transporter and the HAPT1 transport functions. As related in the previous section, HAPT1 was found to be encoded by *TbAQP2* [57] and the MPA resistance was due to a chimeric rearrangement in the *TbAQP2-TbAQP3* locus [55]. Several studies subsequently found such TbAQP2-3 chimeras, or outright TbAQP2 deletions, in clinical isolates from South Sudan and the DRC, demonstrating a clear link between *AQP2* mutations and MPXR [49,62,101]. The definitive word on this was the demonstration that the introduction of a wild-type *AQP2* gene in the resistant *T. b. gambiense* isolates restored sensitivity, whereas the expression of two different chimeric AQP2/3 genes, from an MPXR *T. b. gambiense* isolate and from the MPXR *T. b. brucei* clone B48, into an *aqp2/aqp3* null cell line of *T. b. brucei*, did not [55,102]. *TbAQP2* deletions were also found in two *T. b. rhodesiense* strains adapted in vitro to pentamidine and melarsoprol, respectively [103], and as such there is little remaining doubt, if any, that mutations in the *TbAQP2* gene are the principal determinant of MPXR, and that its unique pore architecture is what made *T. brucei* spp. highly sensitive to them in the first place. Interestingly, the veterinary trypanosome *T. congolense*, which lacks paralogues of both *TbAT1* [104] and *TbAQP2*, is orders of magnitude less sensitive to pentamidine than the *brucei* species. 

## 4. Suramin

While melarsoprol and pentamidine were developed in the 1930s or 1940s, suramin (**16)** is by some distance the oldest trypanocide still in routine clinical use. It was developed out of a series of trypanocidal dyes tested by Paul Ehrlich, starting with Nagana Red (**17**), which displayed only weak trypanocidal properties, followed by the more water-soluble form Trypan Red (**18**) in 1904 [105], which turned out to be both curative and prophylactic for *T. equinum* infections in mice [105,106,107]. As Jim Williamson [7] put it: “This was the classic first cure of an experimentally produced disease by administration of a single dose of a synthetic organic substance of known chemical composition”, and it has had enormous impact on the pursuit of chemotherapy. The 7-amino derivative of Trypan Red was used in a trial in Africa by Robert Koch, but unsuccessfully [107]. Further experimentation with Nagana Red also led to the concepts of acquired drug resistance (‘serum-fast’) in infectious agents and the whole concept of specific targets for different drugs (‘chemo-receptors’) to explain cross-resistance patterns observed [7,108]. However, Nagana Red and its successors, such as Trypan Blue (**19**), all displayed unacceptable side effects at curative doses. Only Trypan Blue was effective in animal models of trypanosomiasis [109,110] and was taken into use as a veterinary drug (against babesiosis), but it stained the meat and skin blue, which did not serve to make it popular and precluded its use on human patients [107]. Further development to an active (and colourless!) trypanocide was undertaken by Maurice Nicolle and Felix Mesnil at the Institut Pasteur [111] in collaboration with by Wilhelm Röhl and Bernhard Heymann at Bayer [7], who via Afridol Violet (**20**), the first of the symmetrical ureas of the series, and after synthesis of >1000 of related structures, found ‘Bayer 205’ in 1916 [112]. This was introduced clinically under the name Germanin, and the formula was kept secret and supplied only to German clinicians, i.e., in German colonial territories [113,114]. As related by Dietmar Steverding [107], the German authorities offered the formula of Bayer 205 to the British Government in return for their lost colonies after World War I, but this was declined. The formula of Germanin was elucidated by Fourneau in 1924 [115,116], and promptly reissued under the name Fourneau 309 [114]. Bayer confirmed the structure 4 years later [107].

For decades now, suramin has only been used for early stage *T. b. rhodesiense* infections, with pentamidine preferred against the *gambiense* disease. There have been few reports of treatment failure with suramin, and it is generally assumed that most of these could have been related to misdiagnosis of cerebral stage HAT. Apted also proposed that in some cases suramin may simply not attain the curative concentration in blood [117], which would constitute treatment failure rather than resistance. However, Pépin and Milord, in their authoritative 1994 review [118], discuss several reports of significant relapse rates in East Africa, and suramin resistance can also be induced experimentally [119], but it is reportedly less stable than tryparsamide resistance, with experimental *T. b. rhodesiense* strains gradually regaining full sensitivity [78].

Suramin has six negative charges at physiological pH and this ensures of course that it will not cross biological membranes unless aided by an active process. One consequence is that suramin must be administered parenterally (i.v. because i.m. and s.c. causes inflammation and necrosis at the injection site [118]) and has no activity against cerebral trypanosomiasis as it is unable to cross the BBB. Another is that this large (MW = 1296 for the free acid, 1429 for the sodium salt), clumsy, un-drug-like molecule that breaks all the Lipinski rules [120], must enter the trypanosomal cell body in order to impact its viability, via an active mechanism. The size and charge of the molecule all but precludes uptake via a nutrient transporter, channel or suchlike. It was proposed that suramin, which binds strongly to Low Density Lipoprotein (LDL) was taken up together with this serum protein by *T. brucei*, via receptor-mediated endocytosis [121,122], but later work, while consistent with uptake via endocytosis, found no correlation between LDL internalization rates and suramin sensitivity [123]. 

As for several other trypanocides, genome-wide screening for loss-of function with an RNAi library in bloodstream form *T. b. brucei* revealed new details of the suramin mode of action and resistance [56]. Most of the hits from this screen concerned its mechanism of uptake, confirming endocytosis, whereas it gave few clues about its mechanism of action, strengthening the view that suramin exhibits polypharmacology. Indeed, suramin has been shown to inhibit many trypanosomal enzymes including dihydrofolate reductase [124], thymidine kinase [125], all the glycolytic enzymes [126] and many others [118,127]. The RNAi screen revealed the suramin receptor to be an Invariant Surface Glycoprotein, ISG75, and also highlighted the involvement of a number of endosomal proteins, lysosomal proteases (Cathepsin L) and a lysosome-based member of the Major Facilitator Superfamily, designated MFST [56]. This has resulted in the current model of suramin uptake, via binding (either free or in complex with a serum protein) to ISG75, delivery to the lysosome by the endosomal system, degradation of the proteinous receptor and finally exit from the lysosome via MFST in to the cytoplasm [127,128]. 

## 5. Eflornithine

Eflornithine (dl-α-difluoromethylornithine; DFMO; **21**) is chemically a close analogue of the amino acid ornithine and pharmacologically a suicide inhibitor of Ornithine Decarboxylase (ODC), i.e., it binds the protein active site irreversibly, via a chemical reaction with a cysteine residue (Cys360 in mouse ODC [129]). ODC is the key enzyme in the cellular production of polyamines (spermine, spermidine, putrescine), which are essential for cell division and as such eflornithine was developed to inhibit cancer cell proliferation [130]. While this was insufficiently effective, due to the very rapid turnover/replacement rate of mammalian ODC (t_1/2_ ~20 min), the drug is currently being investigated by cancer researchers as a chemoprevention agent [131,132]. 

Eflornithine started being tested, with both oral and i.v. formulations, against *gambiense* sleeping sickness from the mid-1980s, with promising results even against late-stage disease [133,134,135,136]. Because it was able to cure melarsoprol-refractory cases and patients already too frail to survive the arsenic-based treatment, it became known as the ‘resurrection drug’ [137]. By the early 2000s, the consensus treatment regimen was established as 100 mg/kg b.w. every 6 h for 14 days, by infusions [138]. However, the treatment was much less successful against *rhodesiense* sleeping sickness than against the *T. b. gambiense* infection [139,140]. The relative refractoriness of *T. b. rhodesiense* was also seen in a test with clinical isolates in mice [141], and Iten et al. [142] concluded that *T. b. rhodesiense* are innately tolerant to eflornithine.

Probably the most important reason that eflornithine worked better against trypanosomiasis than against cancer is that the *T. b. gambiense* ODC is very stable, with a half-life time in excess of 18 h. Thus, the irreversible inhibition of the enzyme by eflornithine ensures that the cell is deprived of polyamines, which it cannot obtain any other way, for a long time (African trypanosomes do not have polyamine transporters, as there are no polyamines in the blood). This seems also to be a main difference with *T. b. rhodesiense* (ODC t_1/2_ ~4.3 h), although the total ODC activity in this species is also higher than in *T. b. gambiense* [141,143]. There was no difference in DFMO uptake between *T. b. rhodesiense* and *T. b. gambiense* [143]. However, several early studies showed reduced eflornithine uptake in resistant cells, which were readily produced in the laboratory [144,145].

One debate [146] with respect to the eflornithine mechanism of action was whether its uptake might be transporter-mediated [145], or by simple diffusion [143,144,147]. This debate has been definitively settled in favour of mediated uptake, as should be expected of a highly soluble, zwitterionic compound with an experimental LogP of −2.7. Vincent et al. [148] induced eflornithine resistance in *T. b. brucei* bloodstream forms and found no mutations in *ODC* but again saw a strongly diminished rate of eflornithine uptake. However, a systematic amplification and sequencing of amino acid transporter genes identified deletions of the gene encoding amino acid transporter 6 (*TbAAT6*) in two independently generated resistant lines. Specific ablation of this transporter by RNAi in a sensitive line resulted in eflornithine resistance, whereas the (re)-introduction of a wild-type *TbAAT6* allele into a resistant strain restored sensitivity [148]. This paper was almost immediately followed by the publication of two other, independent studies using RNAi library screens, which each also identified *TbAAT6* as the main determinant of eflornithine resistance [56,149]. 

While clinical reports about eflornithine resistance in the field are scarce, the fact remains that it is easily induced in the laboratory and that a single point mutation in a non-essential gene (*TbAAT6*) will cause a high level of resistance. Considering that the dosage regimen of eflornithine monotherapy is already severe as well as expensive, and cannot easily be increased in amount or duration, this placed serious question marks over the longevity of the drug, and was a major driver, along with both the cost, duration and logistics of administration, and the adverse effects of 14-day i.v. eflornithine [150], in the development of combination therapies. This included a trial, reported in 2002, of a short treatment with eflornithine followed by three daily injections with melarsoprol, giving a probability of cure of 93% [151]. In 2006, a trial with 54 patients was reported, testing three combinations: melarsoprol-nifurtimox, melarsoprol-eflornithine and eflornithine-nifurtimox [152]. The trial was halted because of the toxicity of the melarsoprol-plus-nifurtimox combination (which also gave only a 44% cure rate), but the eflornithine-nifurtimox performed significantly better than the eflornithine-melarsoprol combination (94.1 versus 78.4%; *P* < 0.05) and resulted in fewer adverse effects. In 2007, two studies, in Uganda [153] and the Republic of Congo [154], described further trials with eflornithine-nifurtimox combination therapy (NECT), which were subsequently extended to a full multi-centre non-inferiority trial [155]. The overall conclusions were that the combination is non-inferior to eflornithine monotherapy and has considerable advantages such as protection against resistance, lower cost, easier and shorter administration as well as a reduction in adverse effects by 50% [155,156]. 

NECT was added to the WHO Essential Medicines list in April 2009 and was adopted as the new first-line treatment for late stage *gambiense* sleeping sickness [156]. At this point, the phasing-out of melarsoprol was considered to be one of the main remaining challenges, as it was still used for ~50% of cases in the Democratic Republic of Congo (DRC), for instance [156]. In 2012, a pharmacovigilance evaluation of 1735 patient outcomes from 9 different countries found that although adverse effects were quite common (60.1%) serious adverse effects were rarely observed (1.1%) and the case fatality rate was 0.5% [157]. This of course compared very well with melarsoprol, the use of which by then had dropped to 12% of all second stage *gambiense* HAT cases [150]. Two further clinical reports, describing NECT outcomes in 684 second stage patients in the DRC [158] and 109 patients in Uganda [159] provided a further evidence base for the selection of NECT as the first-line treatment for cerebral stage infection with *T. b. gambiense*. Based on the low efficacy of eflornithine against *T. b. rhodesiense*, no clinical trials with either eflornithine monotherapy or NECT were initiated against late stage *rhodesiense* sleeping sickness, and to date melarsoprol remains the only approved treatment for that condition. 

## 6. Nifurtimox

Nifurtimox (**22**) has been used since 1969 against *Trypanosoma cruzi* (i.e., Chagas Disease) [160,161] and, given the urgent need to replace melarsoprol for late-stage HAT, has been investigated as a possible treatment for African trypanosomiasis as well [69]. As related by Janssens and De Muynck [162], the first tests with nifurtimox against African trypanosomiasis were conducted by Marc Wéry who found, apparently to everybody’s great surprise, ‘a definite activity on the chronic infection’ in rats, justifying a first trial in humans. That first trial, of just four patients, used 3×120 mg or 3×150 mg daily for 60 or 120 days or however long the drug was tolerated. The trial results were mixed, and it was concluded that nifurtimox was not the sought-after, reliable and low-toxicity replacement of melarsoprol that was hoped for, but that it might serve as a drug of last resort for melarsoprol-refractory patients that were thus untreatable at the time [162]. A subsequent trial in Zaire with 12.5–15.0 mg/kg b.w. daily (in three doses) for 2 months reported cures of 7-out-of-8 melarsoprol-refractory cases and 5-out-of-7 new late-stage cases [163]. In contrast, using essentially the same treatment schedule, Pépin and co-workers reported a relapse rate of 63% in 1989 and concluded that a higher dose would be necessary for cure in most patients [164]. However, after a trial with 30 mg/kg b.w./day for 30 days they concluded that this regimen was ‘significantly toxic’ and that the (only slight) improvement in efficacy did not outweigh the increase in toxicity [165]. Their overall conclusion was that nifurtimox is inferior to eflornithine, then becoming available, as a mono-therapy treatment for arseno-tolerant HAT. Van Nieuwenhove, summarizing the emerging evidence on eflornithine and nifurtimox monotherapy in 1992, strongly advocated trials of combinations of the three available treatments of late-stage HAT [166]. While a treatment regimen starting with melarsoprol alone 2 doses) followed by 10 days of nifurtimox-plus-melarsoprol gave superior cure rates to melarsoprol or nifurtimox alone [167], the combination of eflornithine with nifurtimox (NECT) was eventually adopted, as related in the previous section. 

With the trialling and implementation of NECT, the question of nifurtimox’ mode of action and potential resistance mechanism became one of acute importance. It had long been known that nitro-heterocyclic trypanocides can generate free radicals such as superoxide [168], although direct evidence of this being the principal mechanism of action for nifurtimox and related nitro compounds was lacking. However, the deletion of a gene encoding a glycosomal and cytosolic superoxide dismutase, *TbSODB1*, significantly increased sensitivity to nifurtimox and benznidazole, which was restored to wild-type levels upon re-introduction of the gene; this was specific to *TbSODB1* as deletion of the related glycosomal *TbSODB2* had no effect [169]. Further confirmation of the mechanism of action came with the identification of a mitochondrial NADPH-dependent nitroreductase, NTR, that proved to be essential for the efficacy of a nitro-heterocyclic drug in *T. brucei* and *T. cruzi* [170]. Both species have one copy of this type 1 nitroreductase in their genome. A *T. cruzi* cell line induced for nifurtimox resistance was highly cross-resistant to a variety of nitro-heterocyclic compounds and purified NTR was shown to efficiently reduce all of them. Worryingly, deletion of just one *TbNTR* allele created a heterozygous *NTR*^+/-^ *T. brucei* that displayed no growth phenotype but was three-fold resistance to the nitro-heterocyclic drugs; the double deletion (*ntr*-null) *T. brucei* cell line was even more resistant but also displayed significant growth impairment, leading to the conclusion that the gene is essential [170]. The same gene was subsequently identified in genome-wide RNAi library screens with nifurtimox and benznidazole [171]. As overexpression of *NTR1* resulted in hypersensitivity to the nitro drugs [170] it is clear that *NTR1* is the main determinant of nitro-heterocyclic sensitivity in trypanosomes, and that a single mutation in one allele could eliminate their small therapeutic window, as the main biochemical difference with host cells, as pertains to nifurtimox’ mode of action, is cancelled. 

This conclusion was put to the test by Alan Fairlamb, who induced two nifurtimox-resistant *T. brucei* lines in vitro, which both showed cross-resistance to other nitro-heterocyclic drugs including fexinidazole [172]. The resistant strain displayed unimpeded virulence in mice and, most worryingly, nifurtimox had little effect on the in vivo progression of the infection with this strain [172]. 

The accumulation of nifurtimox across the BBB was investigated by Sarah Thomas using both a murine perfusion system and an in vitro model based on immortalised human BBB cells [173,174]. Nifurtimox readily crossed the BBB and blood–CSF barriers and this was not significantly different in healthy mice or infected mice where the barrier integrity had been compromised [173]. This almost certainly means that the uptake of nifurtimox at the BBB is trans-cellular rather than paracellular (i.e., between cells). Nor was there any difference between the standard model and mice deficient in the BBB efflux transporter P-gp. This was consistent with an earlier study in rats, which found that ^35^S-nifurtimox is rapidly distributed throughout the host, and that both the blood-brain or placental barriers were permeable to the drug [175]. This could be the result of its lipophilicity (octanol-saline partition coefficient = 5.46 [173]), which might allow a simple diffusion across membranes. Certainly, no transporter for nifurtimox uptake has, to date, been identified, either in the host or in trypanosomes, including with RNAi library screens [171]. Yet, nifurtimox, bearing a polar nitro group, is not *very* lipophilic, the trans-cellular uptake at the BBB is consistent with a transporter, and it has been argued that almost all drugs require a transport mechanism [176] and that absence of proof for the involvement of a transporter simply means we haven’t looked well enough [177]. Moreover, Jeganathan et al. reported a higher concentration of ^3^H-nifurtimox in the CNS compared with plasma [173], which would be hard to explain without active import, especially since there is also a component of extrusion across the barrier (see below). Of potential importance for NECT, eflornithine reduced CNS accumulation of ^3^H-nifurtimox by > 50%, presumably by interfering at the level of a transporter at the barrier [173], although that result could not be reproduced in the later study with cultured human BBB cells [174]. If nifurtimox uptake is indeed transporter-mediated, the failure to identify transporter genes with the genome-wide RNAi library could reflect uptake by multiple transporters (non-dependence on a single gene) or the gene being essential (knockdown being lethal). 

Although the existence of a plasma membrane transporter importing nifurtimox has not (yet) been established, Thomas’s studies with the BBB did provide evidence for extrusion across the BBB, despite the non-involvement of P-gp [173,174]. For instance, coadministration with pentamidine enhanced CNS accumulation in the mouse perfusion system, likely indicating an interaction with the ^3^H-nifurtimox efflux transporter at the barrier [173], as also observed with ^3^H-pentamidine distribution [37]. In the follow-on study using immortalised human BBB cells, it was found that accumulation across the barrier was strongly increased by inhibitors of the breast cancer resistance protein (BCRP) but not of P-gp; BCRP is an ATP-dependent efflux transporter of the ABC super family and depletion of ATP in the cell line had the same effect as inhibitors of BCRF [174]. 

Nifurtimox appears to be similarly effective against *T. b. rhodesiense* and *T. b. gambiense*, at least in vitro [178,179]. However, if nifurtimox has been tested clinically on *rhodesiense* HAT, I have been unable to find any reference to it. Certainly, no systematic trials have been held and, considering that NECT is unlikely to work on that infection because of the eflornithine component (see above), the effort would seem almost redundant, and the ethics might be debateable. As such, melarsoprol is still the only approved option for late-stage *rhodesiense* sleeping sickness, although the Drugs for Neglected Diseases initiative (DNDi) started a programme in September 2018 to develop fexinidazole, the newly approved oral drug for late stage *gambiense* disease [180] that does not require co-treatment with eflornithine [181], for the equivalent condition with *T. b. rhodesiense* (https://www.dndi.org/diseases-projects/portfolio/fexinidazole-tb-rhodesiense/). 

As regards fexinidazole, the EMA issued a positive opinion in November of 2018, based on the DNDiFEX004-6 trials, clearing the way for its implementation [182]. However, fexinidazole monotherapy was somewhat less effective than NECT (91.2% vs. 97.6% cure) [181], within the predetermined acceptability margin. However, as pointed out by François Chappuis, this is compensated for by the ease of administration and approved access to medicine that this oral formulation brings to the HAT treatment options [181,183]. Moreover, fexinidazole, was reportedly > 98% curative for early and early-late stage *gambiense* HAT (trial NCT03025789) and can thus be used without having to perform the invasive lumber punctures still required for determination of HAT stage [183]. The most important limitation of fexinidazole, however, seems to be a relatively low curative rate of 86.9% for the patients with the most severe meningoencephalitic HAT (defined as > 100 white blood cells/ml CSF) [1,182]. Thus, for these patients NECT remains the best option [182,184].

## 7. A Perspective on Drug Resistance in African Trypanosomiasis

It is a dogma in the pharmacology of infectious diseases that resistance will (eventually) occur for any drug. Certainly, the infectious agents have many tricks up their proverbial sleeves, often not anticipated [185]. Yet, there is no proof of suramin resistance in African trypanosomes, despite approximately 100 years of usage in East Africa. Additionally, there is no clear proof of pentamidine resistance either, despite intensive use against *gambiense* HAT since the 1940s, including highly successful mass treatments, particularly by the French and Belgian colonial authorities [33]. This even though resistance to either drug can be induced in the laboratory without apparent loss of viability. Thus, there has been relatively little incentive to develop new treatments for the early stage disease and the target product profile of the DNDi has, since its inception, been for late-stage disease. While there was a significant element in this of the need for new treatments with reduced toxicity and cost, leading to optimisation of use of melarsoprol with a shorter 10-day protocol, for instance [83,186], most of the new drug development was driven by resistance to the then-standard treatment. It is questionable whether melarsoprol would have ever been taken into clinical use if not for the catastrophically high levels of resistance with tryparsamide. Similarly, a major factor in the introduction of eflornithine, in addition to melarsoprol’s dangerous toxicity, was the rapid rise in cases refractory to melarsoprol in Central Africa. The well-founded fear of resistance to eflornithine monotherapy (and the anecdotal reports of increasing failures with the drug) helped drive urgent trials with eflornithine and nifurtimox combinations. Every time, we were on our last or only drug against late stage sleeping sickness and the introduction of replacements was sheer necessity.

So, where are we now, in 2020? The number of patients, particularly with *gambiense* HAT, is in steep decline, human-to-human transmission is low, possibly at an all-time low, and we have in hand first-stage treatments that have stood the no-resistance test of time (pentamidine, suramin), and a combination therapy for late stage that is safer than we ever had. The introduction of fexinidazole [181,182,183,184] and potentially acoziborole [2,3], safe oral drugs that are active against both stages of the disease, is finally eliminating the need for risky lumbar punctures for determining the disease stage. The fact that there are finally multiple treatment options would allow clinicians to rotate treatments, should the need arise, but as long as continued vigilance keeps transmission very low, resistance is much less likely to develop. Does this mean sleeping sickness is ‘done’? We have thought this once before, when cases were few, in the 1960s, and have hopefully learned that we must continue the vigilance, awareness and training of medical personnel. More work is also still needed on early detection/diagnosis, detection of asymptomatic cases, animal reservoirs. As far as resistance is concerned, the cross-resistance between fexinidazole and nifurtimox is of potential concern as any strains surviving fexinidazole monotherapy would likely also cause failure with NECT. Thus, the completion of the clinical development and registration of acoziborole and/or melarsoprol-cyclodextrin complex is still of great importance, as is the trial of fexinidazole for late stage *rhodesiense* HAT. Currently Rhodesian HAT is the most neglected disease, still treated with suramin from ~1920 and the awful i.v. melarsoprol in propylene glycol for the late stage. Hopefully one of these new options will finally put suramin to rest and ease this esteemed great-grandfather of chemotherapy into a well-deserved retirement—and i.v. melarsoprol as well.
